# Surgical training in gynecological oncology using soft-embalmed human cadavers: first pilot study

**DOI:** 10.1007/s00404-025-08034-2

**Published:** 2025-04-29

**Authors:** Dariya Jaeger, Eric Hinrichs, Michael Eichbaum, Michael Friedrich, Friedrich-Carl von Rundstedt, Sebastian D. Schäfer, Ralf Schoppe, Sven Schumann, Gebhard Reiss, Georg Feigl, Markus C. Fleisch

**Affiliations:** 1 Institute of Anatomy and Clinical Morphology, University of Witten/Herdecke, Gleiwitzer Strasse 5, 58454 Witten, Germany; 2 Department of Obstetrics and Gynecology, Helios Universitätsklinikum Wuppertal, Wuppertal, Germany; 3 Department of Obstetrics and Gynecology, Helios Klinikum Wiesbaden, Wiesbaden, Germany; 4 Department of Obstetrics and Gynecology, Helios Klinikum Krefeld, Krefeld, Germany; 5 Department of Urology, Helios Universitätsklinikum Wuppertal, Wuppertal, Germany; 6 Department of Obstetrics and Gynecology, Ludgerus Kliniken Münster GmbH, Clemenshospital, Münster, Germany; 7 MoViDo gGmbH, Essen, Germany; 8 Institute of Anatomy, University Medical Center of the Johannes Gutenberg-University Mainz, Mainz, Germany

**Keywords:** Gynaecology, Gynecology, Oncology, Cadaver, Education, Surgical training, Skills training, Anatomy, Soft embalming, Dodge embalming, Thiel embalming

## Abstract

**Context:**

Gynecological oncology requires a high level of surgical expertise. Therefore, new options for realistic surgical training for complex surgical procedures are required. This study aimed to determine how the trainees and experts perceive the use of soft-embalmed cadavers and how realistic it is compared to training on living patients.

**Methods:**

A 2-day hands-on workshop was conducted. Eight trainees were able to practice laparoscopic and open surgical skills on four soft-embalmed cadavers. The training was carried out under the supervision of four gynecological oncology experts, one uro-oncological expert in complication management, and one expert in clinical anatomy. The feedback from trainees and experts was assessed using a structured questionnaire on a 5-point Likert scale. All the procedures were performed in a fully equipped surgical environment and complied with the principles outlined in the Declaration of Helsinki.

**Results:**

The trainees assessed the anatomical and manipulative characteristics of the soft-embalmed cadavers as similar to real conditions. Color and consistency hardly differed from those of the live patients. The trainees stated that soft-embalmed cadavers were beneficial for learning and increased their self-confidence. In terms of realism, all surgical steps were rated a median of 4 out of 5 or higher on a Likert scale by both trainees and experts.

**Conclusion:**

The use of soft-embalmed cadavers for training was perceived positively by trainees and experts. The evaluation results showed a high degree of realism compared with training on living patients. Hands-on training on soft-embalmed cadavers offers great potential as a new training method for surgical skills in gynecological oncology.

## What does this study add to the clinical work

**Table:** 

The use of soft-embalmed cadavers, according to Thiel and Dodge's methods, represents a promising educational approach for realistic surgical training for complex procedures in gynecological oncology.	

## Introduction

Gynecological oncology (GO) requires a high level of surgical expertise. The demand and significance of structured education of young surgeons is further highlighted by the findings of numerous studies that indicate a correlation between the level of surgical training and postoperative outcomes [1, 2]

The high degree of complexity involved in these surgical procedures requires the implementation of comprehensive training concepts that incorporate various educational methodologies. The European Society of Gynecological Oncology (ESGO) has developed a curriculum to standardize training and enhance the preparedness of surgeons to undertake complex procedures such as cytoreductive surgery for ovarian cancer [3]. This initiative demonstrates a broader recognition of the necessity for comprehensive training programs that encompass both surgical skills and multidisciplinary collaboration, which are essential for managing the complexities associated with gynecological malignancies [3, 4].

An innovative approach is the use of soft-embalmed human cadavers. Research findings indicate that cadaveric dissection workshops significantly improve the surgical anatomical knowledge of gynecological oncology fellows. Furthermore, the participants reported high levels of satisfaction and perceived value from such training experiences [5].

Laparoscopic techniques and surgical anatomy workshops are essential for trainees, providing hands-on experience between theoretical knowledge and practical application [2].

Soft-embalmed cadavers according to Thiel [6] offer advantages over traditional formalin-preserved specimens [7], including better preservation of tissue texture and color, which facilitates a more realistic learning environment [7, 8]. Soft-embalmed cadavers according to Dodge (Dodge Solutions, Dodge Co., Billerica, MA, USA) also exhibit a high degree of realism, flexibility, and a high degree of realism in the colors of fresh tissue [9, 10].

Surgical training on Thiel embalmed cadavers (TeCs) provides an effective and realistic training option for challenging surgical procedures, which has already been demonstrated in previous studies [11–13]. To the best of our knowledge, there is only one published study on the use of Dodge embalmed cadavers (DeCs) in surgical workshops [10]. To date, there have been no reports of the use of DeCs in GO. 

The objective of this study was to provide a detailed account of our experiences with the inaugural pilot study utilizing soft-embalmed cadavers in accordance with Dodge’s and Thiel’s methods for surgical hands-on training in GO, including laparoscopic and open surgical techniques. Additionally, we aimed to ascertain the training experiences of trainees and experts regarding realism and special aspects of training on soft-embalmed cadavers as educational concepts.

## Material and methods

### Soft-embalmed human cadavers: Thiel’s and Dodge’s methods

Four soft-embalmed cadavers were used during training. Two cadavers were preserved using the Thiel method, and two cadavers were preserved using the Dodge method (Dodge Solutions, Dodge Co., Billerica, MA, USA). TeCs were donated to the Department of Anatomy at Gutenberg University of Mainz and subsequently transferred to the University of Witten/Herdecke for use in postgraduate courses following the completion of the embalming process. DeCs were donated to a non-profit prosecture MoViDo gGmbH placed in Essen, Germany, and given to the University of Witten/Herdecke for scientific examination for this study after the embalming process was completed.

Human cadavers were used under the strict rules of the donation programs of the Department of Anatomy at the Gutenberg University of Mainz and MoViDo gGmbH. The use of the cadavers was reviewed with the positive vote of the local ethics committee of the University of Witten/Herdecke.

Both soft-embalming methods create soft and flexible cadavers with very good color contrasts of the tissue and almost realistic lifelike conditions for surgeons [6, 9]. Therefore, TeCs and DeCs can be used for training by many different clinical disciplines [11, 12, 14].

### Selection of the human cadaver

The selection of cadavers was based on the female sex and the integrity of the surgical field to avoid previous organ resections of the target organs (e.g., uterus, ovaries, etc.) of the planned surgical procedures. In this case, the selection process entailed the exclusion of surgical scars in the affected abdominal region. No further information was available on the cadavers'previous illnesses or surgeries owing to data protection regulations for body donation.

The cadavers used for training did not indicate prior abdominal surgeries. Intraoperatively, during training, previous diseases of the target organs could be excluded macroscopically.

### Selection of the trainees

The candidates were required to provide details of their surgical experience, current professional position, and the clinic in which they were employed. The previous surgical experience of the participants was a significant factor in their acceptance by course organizers. A prerequisite for consideration was the desired or existing specialization in GO.

### General logistics

The training was organized as a 1.5-day-workshop including half a day as a lecture part and a whole day as a hands-on part.

Eight participants performed laparoscopic and open-surgery procedures on four soft-embalmed cadavers under the supervision of four board-certified gynecologic oncologists as experts, one expert in urological oncology, and one expert in clinical anatomy.

Each GO expert supervised a team of two trainees. The setting was created to reflect the conditions typically found in an operating room, with original surgical instruments for minimally invasive and open surgery, suture materials, and other surgical consumables.

The workshop was organized at the UMBILICUS Skills Lab of the Institute of Anatomy and Clinical Morphology of the University of Witten/Herdecke, Witten, Germany. The objective of the workshop was to provide postgraduate surgical trainees with high-quality, hands-on educational experience on soft-embalmed cadavers using Thiel’s and Dodge’s methods. The cadavers were supplied by the Anatomical Institute of the University of Witten/Herdecke in collaboration with the Anatomical Institute of the University of Mainz and MoViDo gGmbH.

### Surgical logistics

**Fig. 1:**
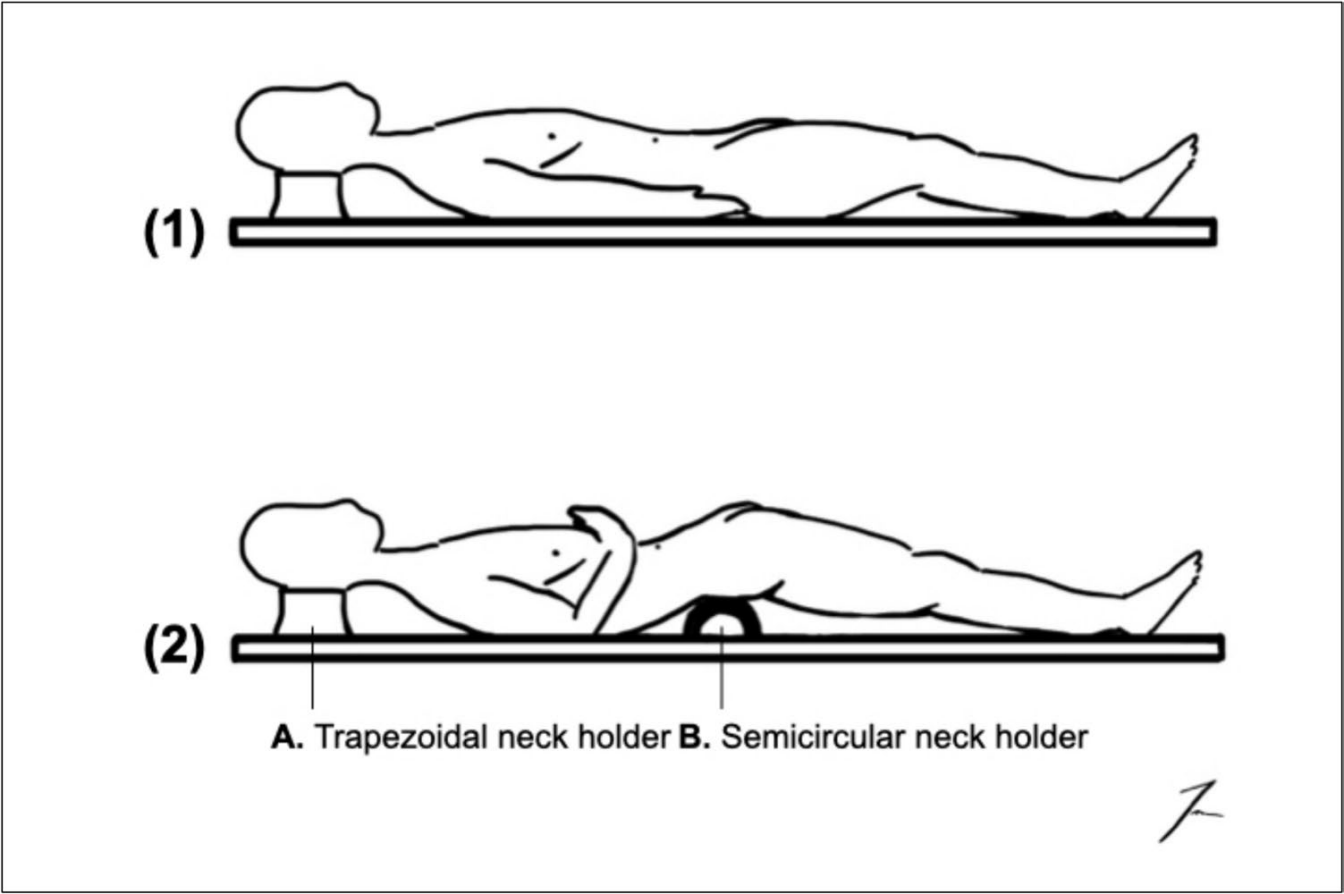
The following scheme illustrates the positioning of the cadaver [11]: (1) The cadaver was positioned on the anatomical preparation Table (2) The use of a semicircular neck holder **B** made it easier to lift the lower abdomen. The right arm was abducted, facilitating a more comprehensive visualization of the semicircular neck holder. In Figs. 1 and 2, the head is positioned on a standard trapezoidal neck holder **A** that can also be used for placement

Hands-on training was conducted in the standard dissection room at the Anatomical Institute of Witten/Herdecke. The regular anatomy preparation tables were repurposed as the operating tables for the workshop. The tables were not adjustable and could not be changed in height or tilted to the side. Positioning maneuvers of the cadaver could be carried out manually, for example, by placing a semicircular neck holder underneath the lower back to optimize the view of the pelvis, as shown in Fig. 1.

**Fig. 2:**
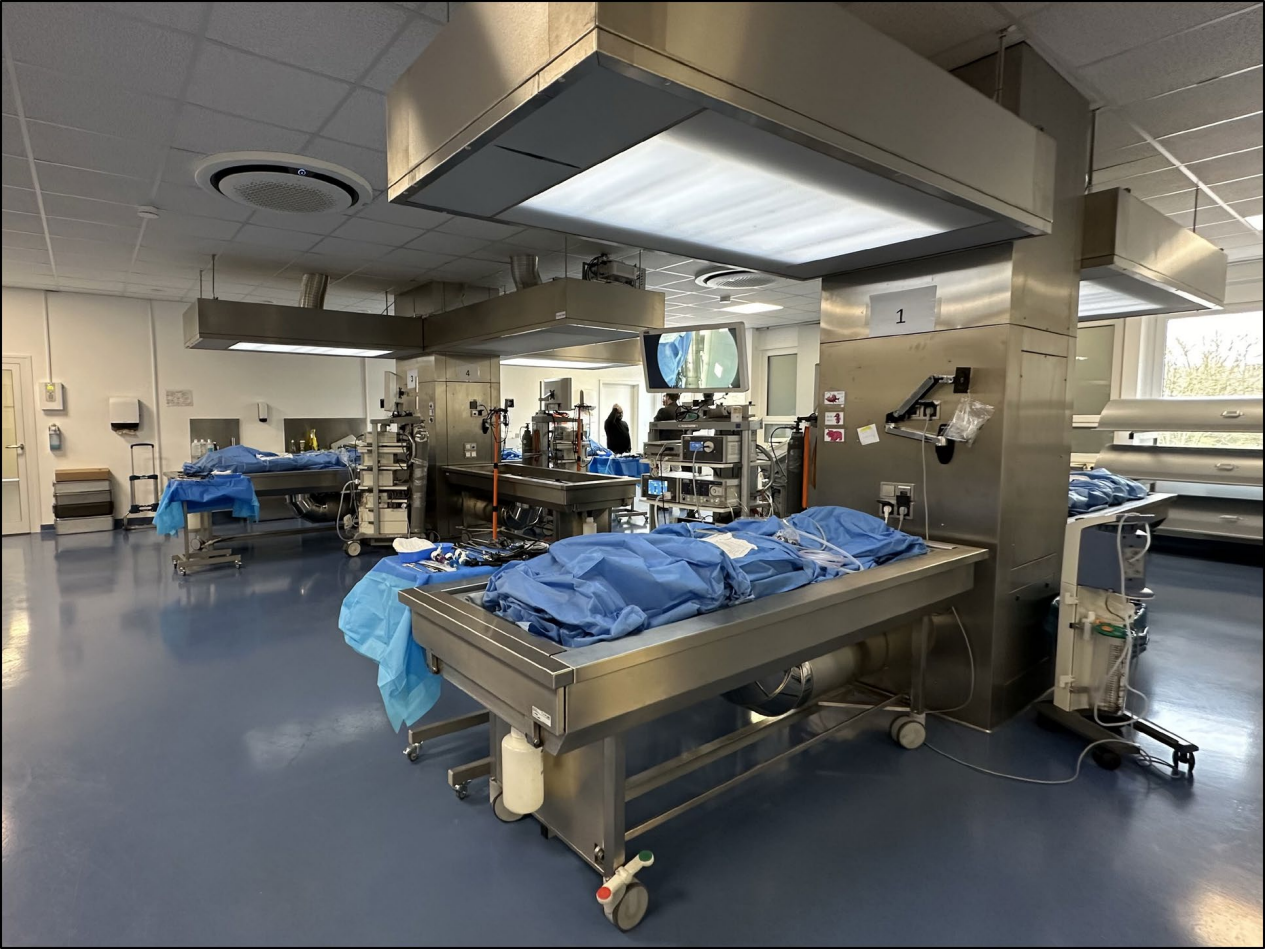
Overview of the anatomical dissection hall during the training

**Fig. 3:**
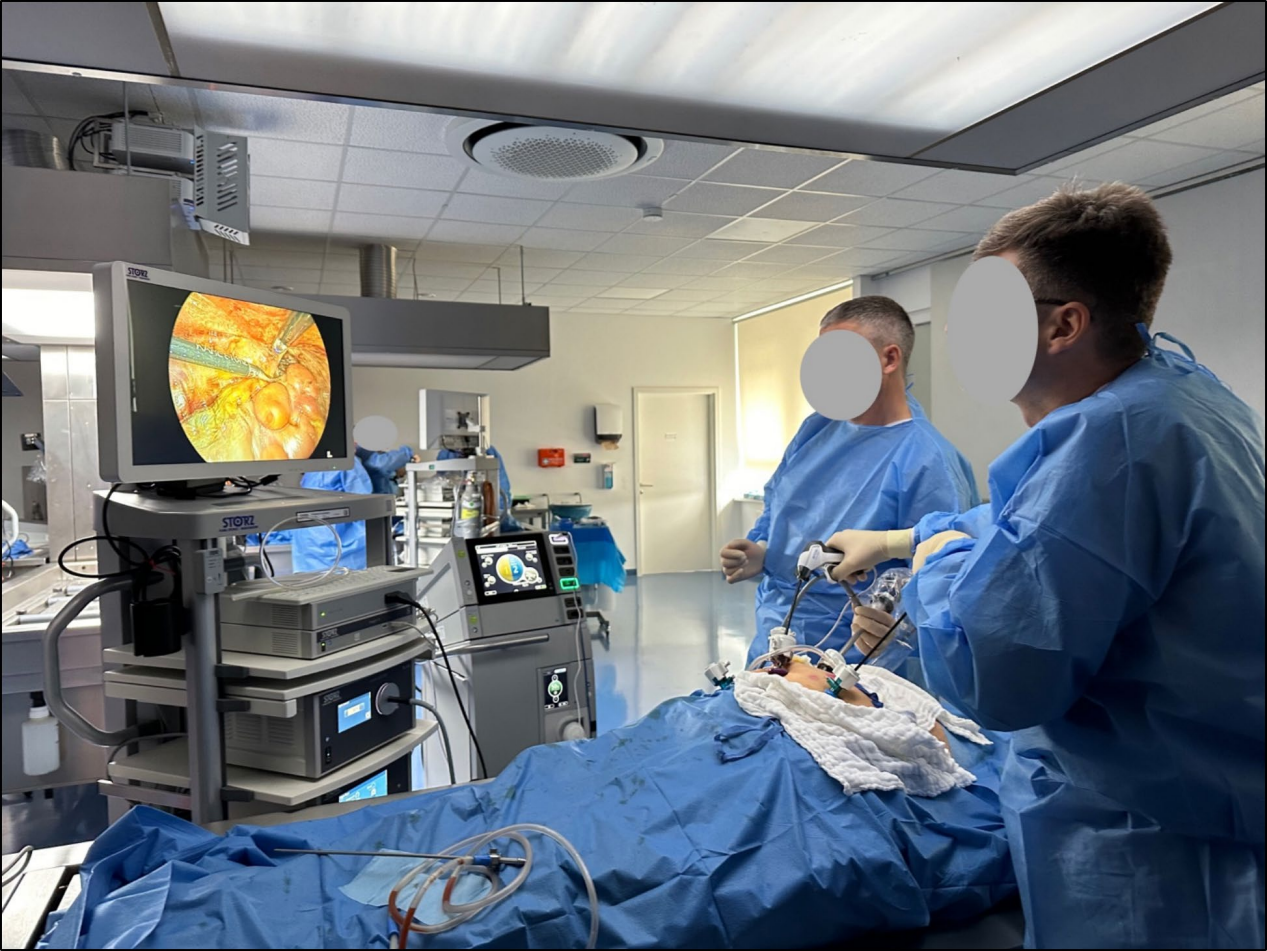
Trainees during minimally invasive surgery

Figures 2 and 3 show the settings during training. A powerful lighting system was fixed above the tables, covering their entire length. Additive freestanding focusing lights were used for the deep dissection of the pelvis. The training utilized the original surgical instruments for laparoscopic and open surgery, in addition to the original endoscopic systems and energy devices provided by sponsors (Fig. 2). For the open surgery component of laparotomy, the original wound retractors were employed to facilitate a realistic overview of the situs.

### Surgical technique

After completing the theory on days 1 and 2, hands-on training was continued on soft-embalmed cadavers. The structure of the hands-on training was modular. Starting with minimally invasive surgery and moving to the open surgery module.

All surgical procedures were conducted in accordance with standard protocols, typically performed in the operating room on a living patient. All surgeries were performed by trainees under the supervision and guidance of an expert. The trainees alternated between the roles of the surgeon and first assistant.

Hands-on training started with diagnostic laparoscopy and laparoscopic and oncological systematic para-aortic and pelvic lymphadenectomy as part of a minimally invasive surgery. After completion of lymphadenectomy, multivisceral resection was performed, including the management for intestinal lesions and lesions of the urinary tract. Reconstruction techniques for the ureter or treatment of bladder wall injuries were demonstrated and supervised by an uro-oncological expert. Finally, extraperitoneal preparation for radical hysterectomy was performed. 

All procedures were conducted under the supervision of a clinical anatomy expert, whose expertise was employed to facilitate comprehension of the complex anatomy of the pelvic area, particularly that of the small pelvis and the pelvic floor.

### Evaluation

A structured questionnaire was developed in accordance with the model proposed by Jaeger et al. [11]. To this end, a modular structure for the questionnaire was devised in collaboration with the workshop director, a chief physician and board-certified gynecological oncologist with over 25 years of professional experience, based on the planned educational surgical procedures for the workshop. The objective of the questionnaire was to compare all essential surgical procedures and steps in terms of their realism when performed on a living patient.

A questionnaire with 25 questions in four modules was presented for anonymous subjective feedback from trainees and experts. The questions were rated on a 5-point Likert scale (1 = ‘strongly disagree’ to 5 = ‘strongly agree’).

The questionnaire contained modules A = general questions about the training experience (e.g. general realism, recommendation), B = realism during the surgery (e.g. tissue texture, haptics, anatomy, etc.) and special aspects of the training in module C (e.g. ethical aspects and lack of bleeding) and module D (e.g. surgical teaching and lack of time pressure).

An overview of the questionnaire is shown in Table 1. The originally used questionnaire is presented in the supporting Information of the article as Table S1.

**Table 1:** Used questionnaire including the modules A–D

Module A	General questions about the training experience
1	Performing the surgery on soft-embalmed cadavers provides good preparation for performing the surgery on a live patient
2	Hands-on training of the performed surgical procedures on soft-embalmed cadavers under supervision could make inexperienced surgeons more confident in carrying it out
3	I felt that the procedure on the soft-embalmed cadaver was realistic
4	The implementation of this hands-on training under the supervision of a training supervisor should be creditable towards the further training catalogue
5	I can recommend the performed hands-on training in form of a workshop

Module B	Specific questions about the feasibility and realism of the surgery on soft-embalmed cadaver
1	Surgical access/laparotomy was realistically possible
2	The anatomy and feel of the tissue were realistic
3	The consistency of the uterus and the adnexa showed hardly any differences to live patients
4	The retroperitoneal anatomy on the cadaver allowed the usual orientation
5	The dissection of lymphatic vessels, arteries and veins was realistically possible except for the lack of bleeding
6	Inspection reveals clearly recognizable tissue layers for layer-appropriate preparation
7	The tissue is soft and elasticated
8	The tissue layers can be easily moved against each other
9	The preparation of the tissue offers a realistic surgical experience

Module C	Special aspects of training on soft-embalmed cadavers (Ethical aspects and lack of hemorrhage)
1	Operating on an already deceased human cadaver reduced my concern and the associated ethical pressure of creating possible surgical complications
2	Due to the lower ethical pressure from possible surgical complications with an already deceased human cadaver, I carried out the individual surgical steps more confidently
3	Operating on an already deceased human cadaver reduced my anxiety and the ethical pressure of creating possible surgical complications
4	Due to the lack of fear of creating possible bleeding complications, I carried out the individual steps of the operation more confidently
5	Due to the lack of bleeding during surgery, I was made particularly aware of the possible sources of bleeding and pitfalls by the expert
6	Due to the lack of bleeding during surgery and the specific indications of the possible sources of bleeding by the expert, I became even more aware of the possible sources of bleeding
7	I found the lack of bleeding during surgery training to be a disadvantage
8	I found the lack of bleeding during surgery training to be an advantage

Module D	Surgical teaching/lack of time pressure
1	As there was no time pressure, I was able to concentrate better on carrying out the individual steps of the operation
2	The lack of time pressure meant that I was able to concentrate better on teaching during the operation
3	In view of the lack of time pressure, I had the opportunity to answer more of the participants'questions
4	In terms of the lack of time pressure, I found surgical teaching under supervision to be more effective—compared to training on living patients

## Results

Feedback from the trainees and experts was analyzed using a comprehensive questionnaire with a 5-point Likert scale (1 = ‘strongly disagree’ to 5 = ‘strongly agree’). The questionnaire contained the modules A = General questions about the training experience (e.g., general realism of the training, recommendation), B = Specific questions about the feasibility and realism of the surgery on soft-embalmed cadavers (e.g., tissue texture, haptics, anatomy, etc.), Module C included special aspects of training on soft-embalmed cadavers (e.g., ethical aspects and lack of bleeding), and Module D included questions about the impression of the surgical teaching and the lack of time pressure during the training. The evaluation did not include any measures to verify the effectiveness of the surgical skills training provided during class.

A total of 12 questionnaires were analyzed, four of which were completed by GO experts and eight by trainees.

### Results from experts and trainees

**Fig. 4:**
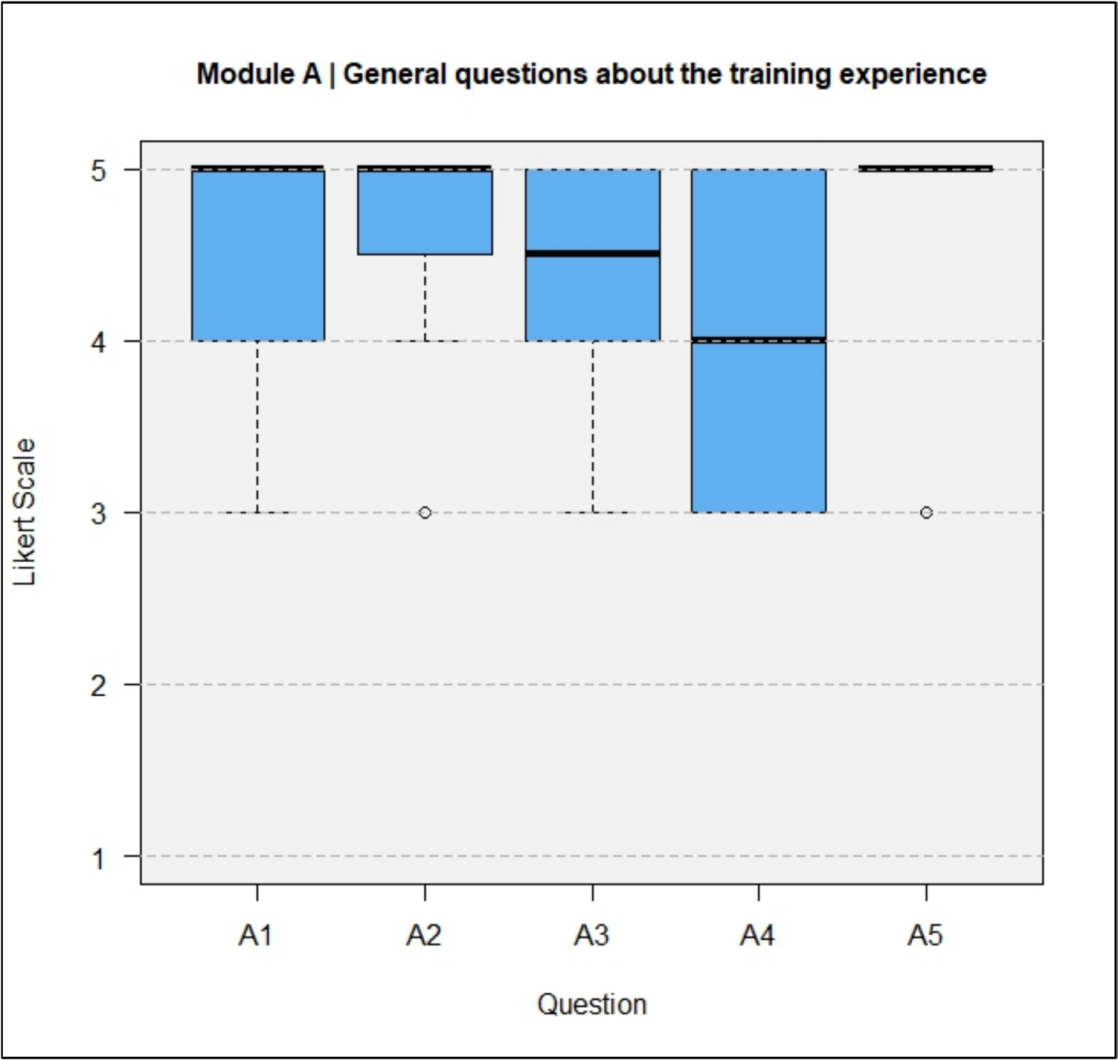
Module A, General questions about the training experience

**Fig. 5:**
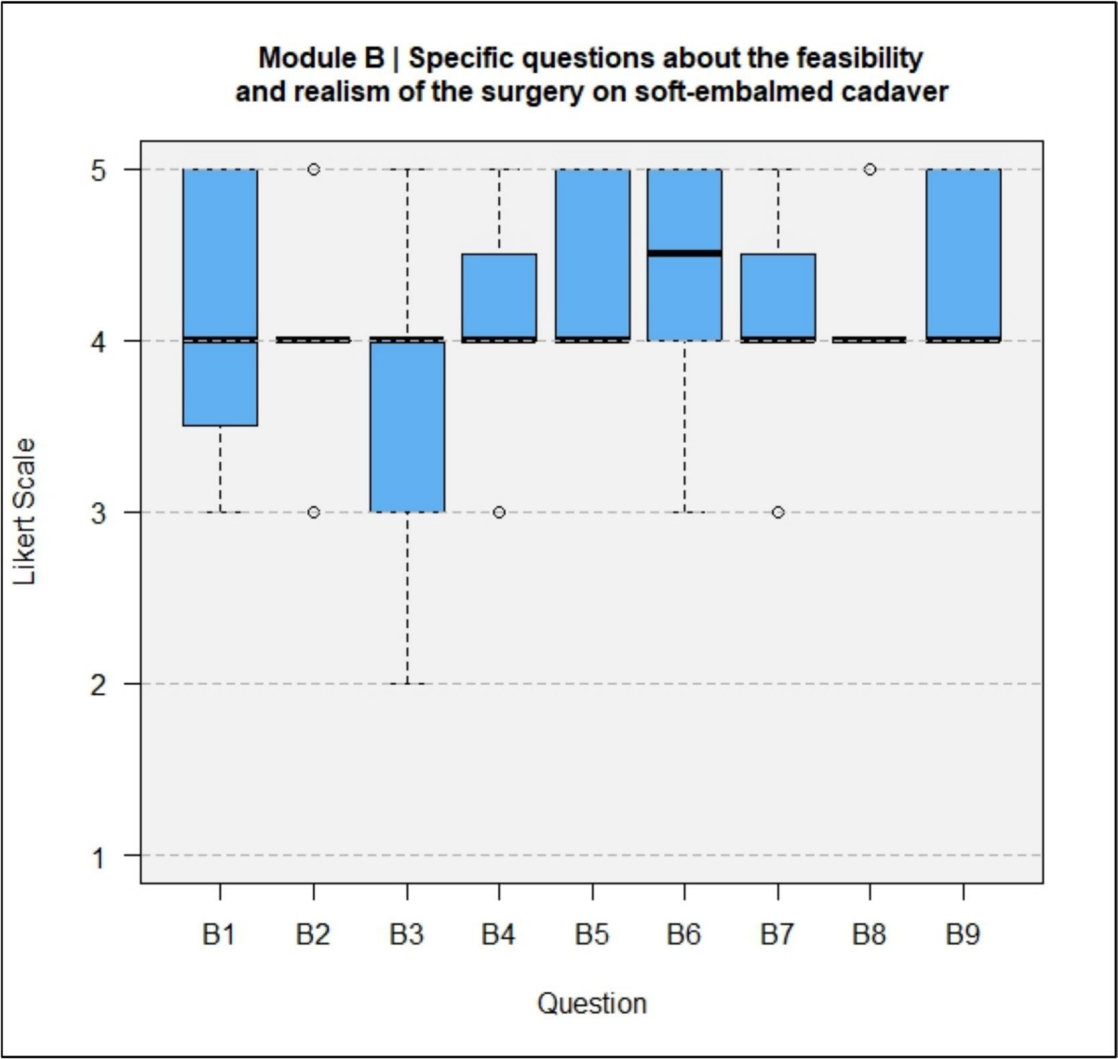
Module B, Specific questions about the feasibility and realism of the operation on soft-embalmed cadaver

**Fig. 6:**
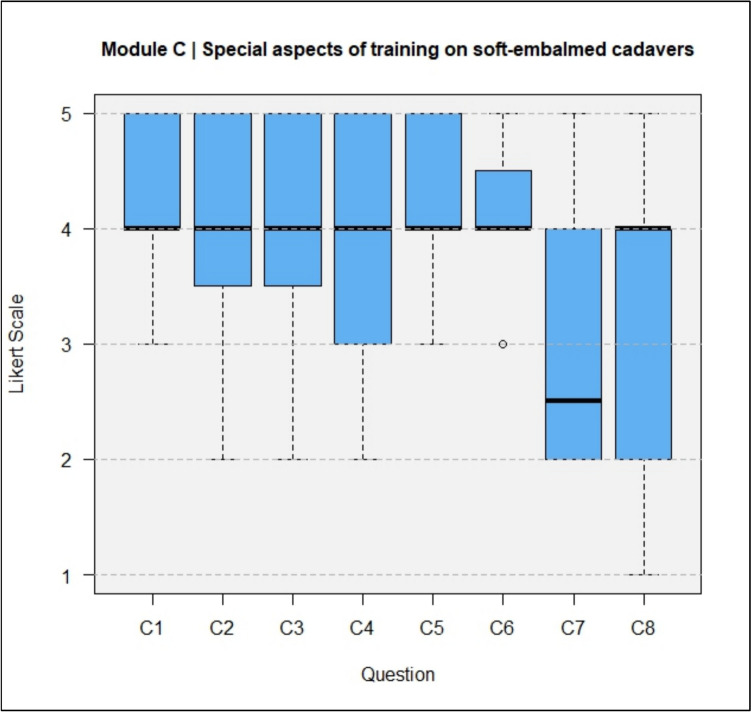
Module C, Special aspects of training at soft-embalmed cadavers

**Fig. 7:**
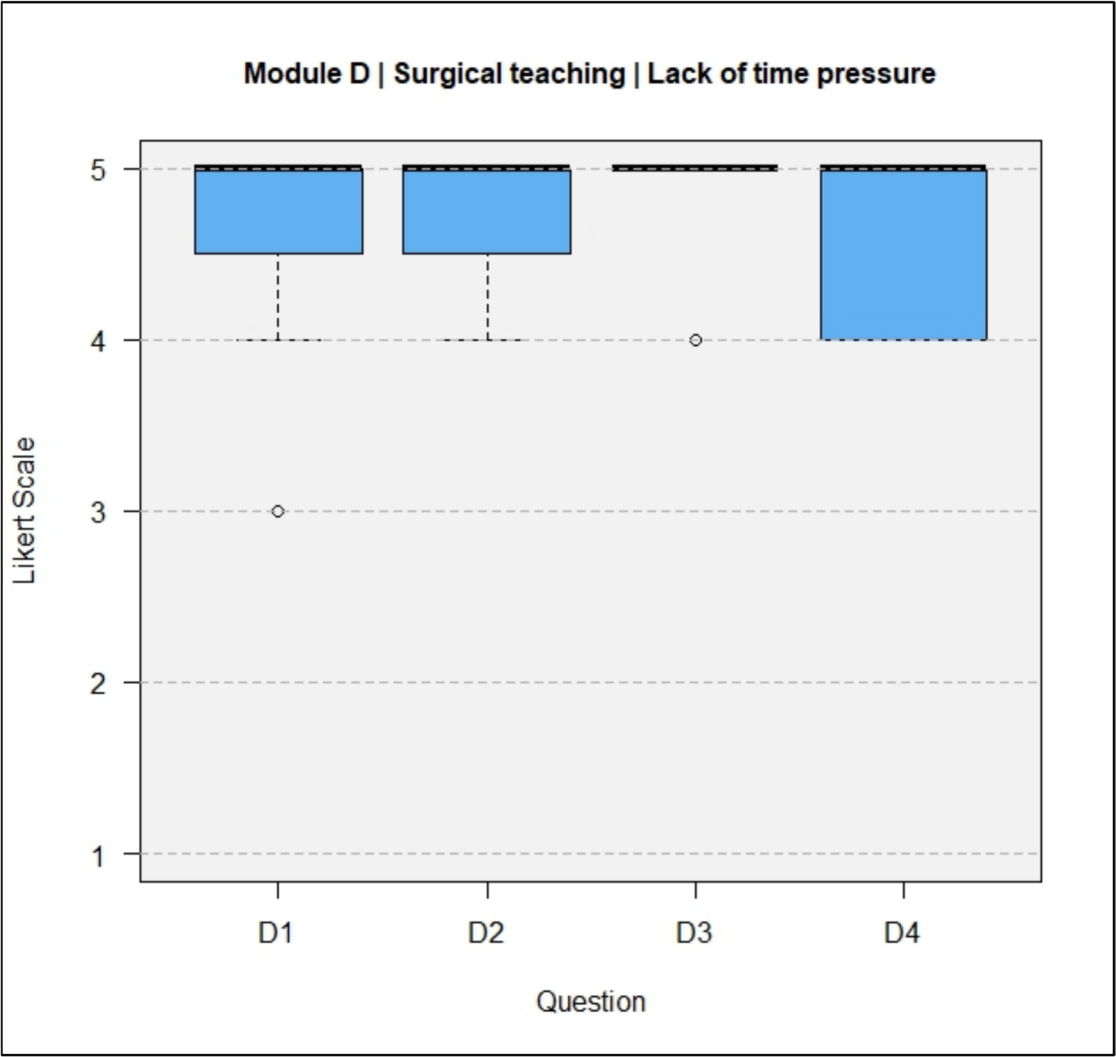
Module D, Surgical teaching/Lack of time pressure

The results of the questionnaires including both types, DeC and TeC, are shown in full in Figs. 4, 5, 6, 7.

All questions for TeCs and DeCs regarding the realism of cadaveric anatomy (B2) were rated with a median of 4 (agree) by trainees and experts.

The training was rated as realistic (A3, median = 4.5) and as good preparation for live surgery (A1, median = 5). Training under supervision could make inexperienced surgeons more confident in performing the operations (A2, median = 5).

In Module B, all specific questions about the feasibility and realism of the operation on a soft-embalmed cadaver were answered with a median score of 4 or better. The anatomy and feel of the tissues were rated as realistic (B2, median = 4).

The lack of concern about possible surgical complications in body donors reduced the ethical pressure to avoid causing intraoperative complications (C1, median = 4). Trainees and experts disagreed (C7, median = 2.5) that a lack of bleeding was perceived as a disadvantage. The lack of bleeding was perceived as an advantage of training (C8, median = 4).

Regarding the lack of time pressure during training, surgical teaching under supervision was perceived as more effective than training on live patients (D4, median 5). At least, surgical training in the form of a workshop was rated as recommendable (A5, median = 5).

### Comparison: Dodge vs. Thiel

**Fig. 8:**
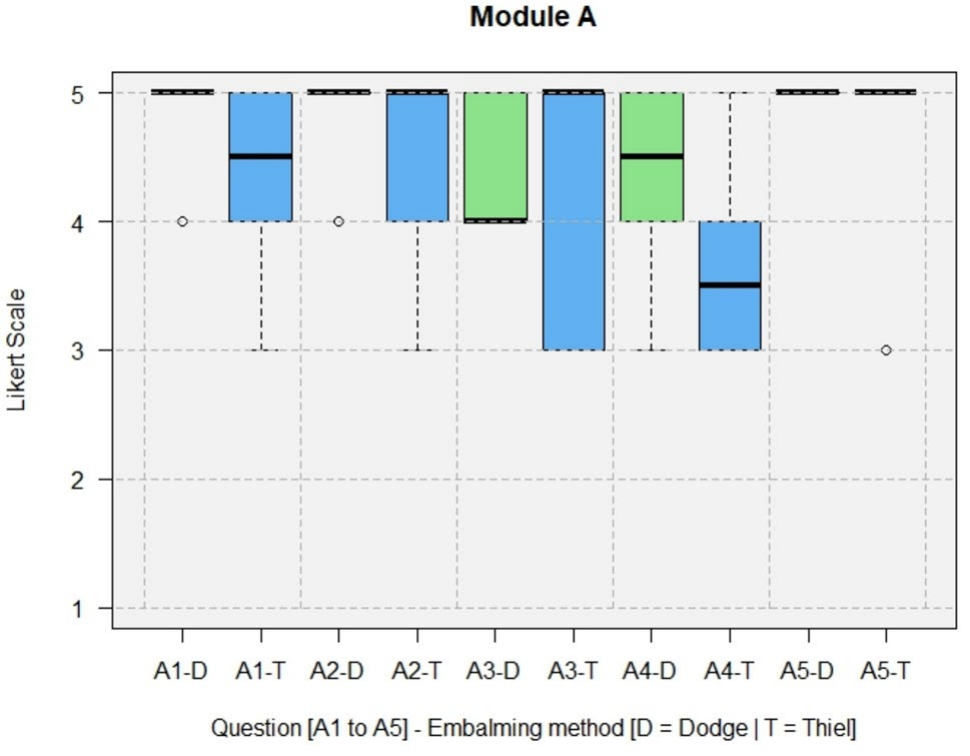
Module A, comparison of the results between DeCs and TeCs

**Fig. 9:**
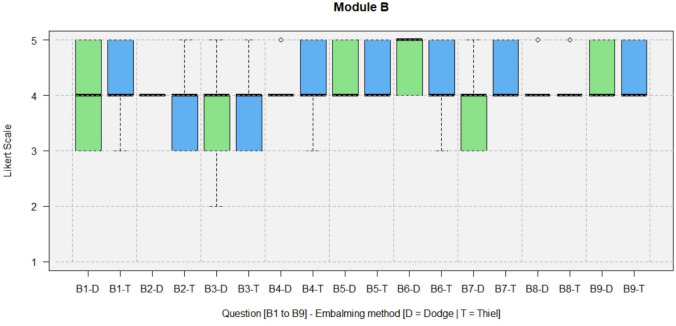
Module D, comparison of the results between DeCs and TeCs

A comparison of the results of the DeC and TeC questionnaires is shown in Figs. 8, 9. The differences in the boxplot quartiles between DeCs and TeCs can also be viewed here.

In Module A (Fig. 8), performing the surgery on soft-embalmed cadavers as a good preparation for performing the surgery on a live patient (A1) was rated with a median of 5 for DeCs and with a median between 4 (agree) and 5 (strongly agree) for TeCs. The training was rated as realistic (A3) with a median of 4 (agree) for DeCs and 5 (strongly agree) for TeCs. The implementation of supervised hands-on training as creditable for a surgical catalogue (A4) was rated with a median 4 (agree) to 5 (strongly agree) for DeCs and with a median 3 (neutral) to 4 (agree) for TeCs.

For DeCs and TeCs, a median of 5 confirmed that hands-on training of surgical procedures performed on soft-embalmed cadavers under supervision could give inexperienced surgeons more confidence in performing them (A2). For DeCs and TeCs, the median rating for recommending hands-on training in the form of a workshop (A5) was 5 (strongly agree).

**Fig. 10:**
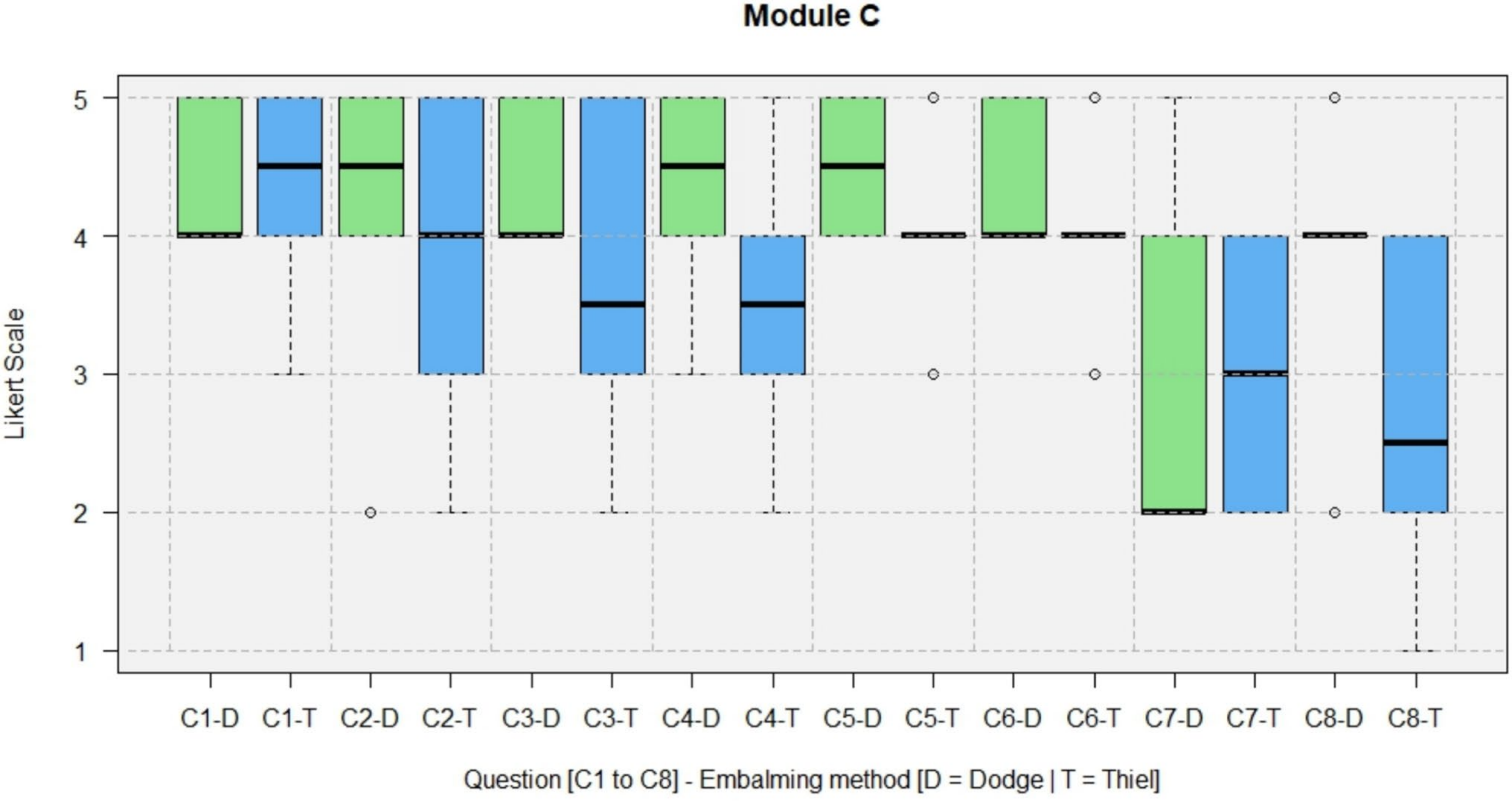
Module B, comparison of the results between DeCs and TeCs

In Module B (Fig. 10), all specific questions about the feasibility and realism of the operation on soft-embalmed cadavers were answered with a median of 4 (agree) or better for DeCs and TeCs. In terms of inspection and clearly recognizable tissue layers for layer-appropriate preparation (B6), the median for DeCs was 5 (strongly agree) and the median for TeCs was 4 (agree).

**Fig. 11:**
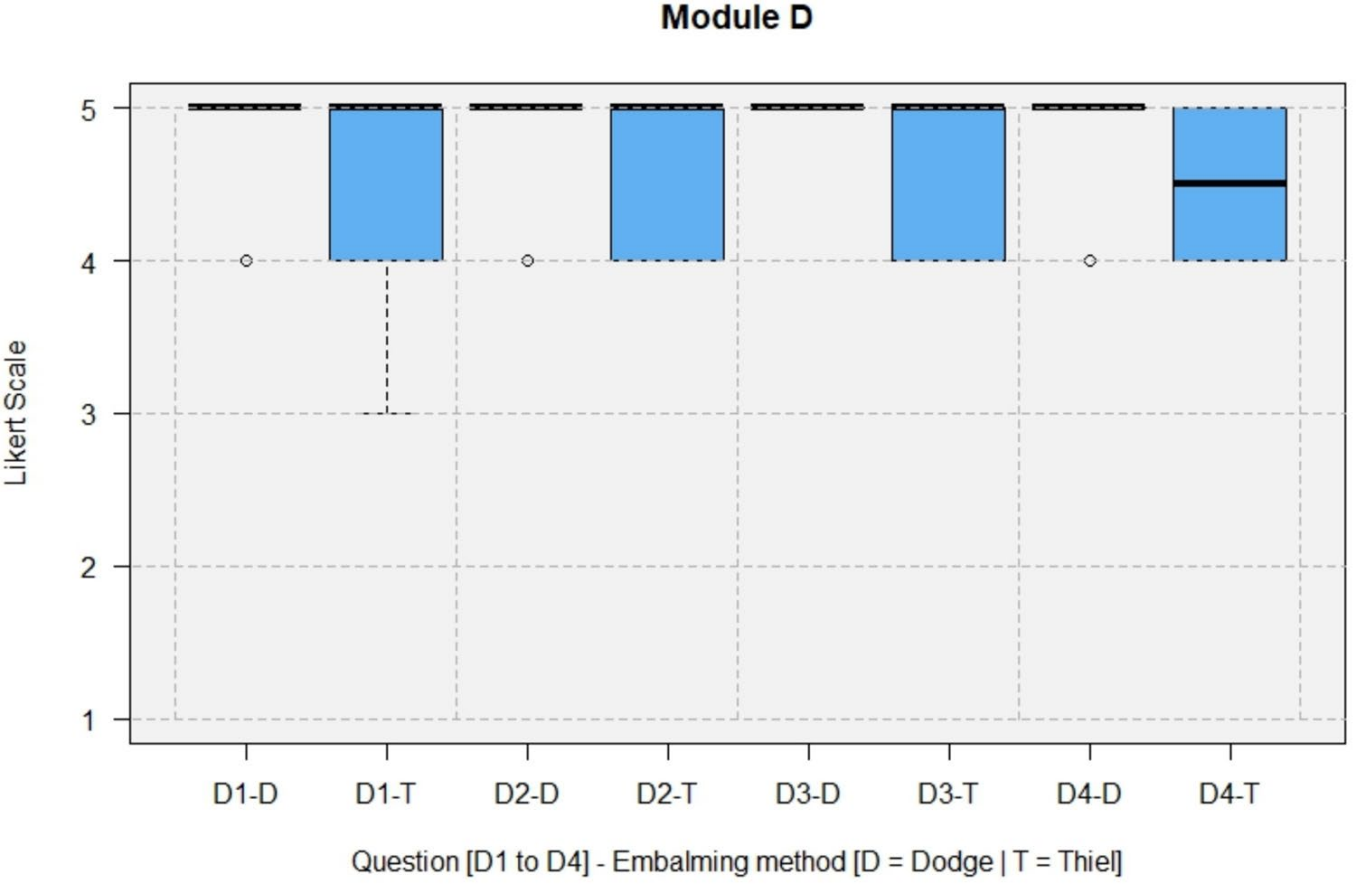
Module C, comparison of the results between DeCs and TeCs

In Module C, differences in median scores between DeCs and TeCs can be seen (Fig. 11). The question about whether performing surgery on an already deceased human cadaver reduces anxiety and ethical pressure about possible surgical complications (C3) was rated with a median of 4 (agree) for DeCs and a median of 3 (neutral) to 4 (agree) for TeCs.

The question of whether the lack of bleeding during surgery training was found to be a disadvantage (C7) was answered with a median of 2 for DeCs and a median of 3 (neutral) for TeCs. The question of whether the lack of bleeding during surgery training was found to be an advantage was answered with a median of 4 (agree) for DeCs and a median of 2 (disagree) to 3 (neutral) for TeCs.

In Module D, the questions for DeCs and TeCs were answered with a median of 5 (strongly agree) for all questions except D4, which had a median of 4 (agree) for DeCs and a median between 4 (agree) and 5 (strongly agree) for TeCs.

## Discussion

The necessity and importance of comprehensive surgical education for young surgeons in GO is becoming increasingly evident, demonstrating a correlation between surgical training and postoperative outcomes for the patients [1, 2].

As the field of GO continues to grow, it is essential that the next generation of gynecological oncologists receive training that incorporates a variety of methodologies. This will ensure that they are equipped with the skills and knowledge necessary to provide excellent care to patients. The training methods that are becoming increasingly important in this field include the use of soft-embalmed cadavers, laparoscopic workshops, and robotic surgery courses.

Workshops focusing on laparoscopic techniques and surgical anatomy are crucial element of the training process [2]. This highlights the need for hands-on training concepts that effectively combine theoretical knowledge and practical applications in complex anatomical situs.

Furthermore, there has been a notable increase in the incorporation of robotic surgery training into fellowship programs, reflecting the growing prevalence of robotic techniques in GO [15, 16]. Such training has two benefits: it enhances technical abilities and prepares participants for the evolving landscape of surgical oncology, where minimally invasive techniques are often preferred because of their benefits, including shorter recovery times and lower complication rates [17].

To our knowledge, there are currently no publications on the use of soft-embalmed cadavers according to Thiel and Dodge in hands-on training for robotic surgery in GO. Conceptual planning of these is already underway.

This study presents the inaugural experience of GO training on soft-embalmed cadavers using Dodge’s method in comparison with Thiel’s cadavers. The evaluation results showed a high degree of realism compared to surgery on living patients, as rated by the trainees and experts. The anatomical orientation and tissue characteristics were rated realistic. Due to the lack of time pressure during training, surgical teaching on cadavers was perceived to be more effective than training on living patients.

The results offer a promising new method of surgical training for complex procedures in GO for both embalming methods. Nevertheless, certain limitations were considered in this pilot study.

First, larger sample sizes and further studies are needed to verify the results of this study and enable extended statistical analyses to show significant differences between TeCs and DeCs. The feedback from the participants is valuable for the first experience reports of surgical training of GO on soft-embalmed cadavers, but it is based on subjective experience. A larger number of participants and objective measures are necessary for future studies.

Second, the questionnaire should be extended by further modules, for example, to detect possible inter-individual differences in preservation quality between cadavers. Despite following the standard protocol for embalming, there may be differences in the coloring or texture (softer/harder) of the tissue due to individual cadaver variability or a significant difference between Thiel’s and Dodge’s embalming methods. These aspects could influence the impressions of the trainees and experts regarding the realism of the training experience on cadavers and should be included in the questionnaire.

Furthermore, the comprehensive but informal questionnaire was limited to training experience within the predefined questionnaire. For instance, the inclusion of (video-) interviews could incorporate new aspects from the experience reports of trainees and experts that had not been previously considered.

Third, our study did not include any objective measures to verify the effectiveness of the surgical skills training provided during class. The pre- and post-training assessments or the use of a comparison group (e.g., dry-lab skills training vs. cadavers) could improve educational significance and objectivity of the findings.

A follow-up study focusing on the learning success of surgical training in clinical applications, such as the transfer of skills into the operating room to living patients, would provide valuable insights and contribute to the wider field of surgical education. However, it will be highly beneficial to investigate the learning success of training programs in future studies with larger sample sizes.

Should future research confirm a clear educational benefit of soft-embalmed cadavers in surgical training in GO, the next step would be to incorporate hands-on practice into surgical fellowship curricula in GO. This approach would enable inexperienced surgeons to learn complex surgical procedures first on a cadaver in a controlled environment before they are permitted to perform them independently on GO patients, who are typically critically ill and require the best possible care.

A further imperative step is a detailed comparison of the two soft embalming methods. In addition to potential tissue variations, factors influencing cost efficiency, availability, preservation time, storage, and other logistical challenges should be incorporated into the comparison. Conducting such an investigation would necessitate a separate extended study.

## Conclusion

Surgical training on human cadavers using the Thiel and Dodge method of soft embalming was highly recommended by trainees and experts of the GO. The evaluation results indicated a high degree of realism compared to surgical procedures performed on living patients for both embalming methods*.*

The use of soft-embalmed cadavers represents a promising educational approach for surgical training of complex procedures in GO. Further studies with larger sample sizes are required for significant results to confirm the findings.
